# Supplementary material for: The Characteristic of HBV Quasispecies Is Related to Occult HBV Infection of Infants Born to Highly Viremic Mothers
Source: Viruses. 2024 Jul 9;16(7):1104. doi: 10.3390/v16071104 (PMC11281566; doi:10.3390/v16071104)
Supplement: Supplementary file 1 [file viruses-16-01104-s001.zip › viruses-3041516-supplementary.pdf]

The Characteristic of HBV Quasispecies Is Related to Occult HBV Infection of Infants Born to Highly Viremic Mothers

Yi Li[1,2,3,†], Yarong Song[1,†], Yiwei Xiao[1], Tong Wang[2], Lili Li[1], Minmin Liu[1], Jie Li[1,*], and Jie Wang[1,4*]

Author Affiliations:

[1]Department of Microbiology & Infectious Disease Center, School of Basic Medical Sciences, Peking University Health Science Center, Beijing 100191, China

[2]Department of Clinical Laboratory, Peking Union Medical College Hospital, Chinese Academy of Medical Sciences and Peking Union Medical College, Beijing 100730, China

[3]Graduate School, Peking Union Medical College, Chinese Academy of Medical Science, Beijing 100730, China

[4]NHC Key Laboratory of Medical Immunology, Peking University, Beijing 100191, China

*Correspondence: jieli@hsc.pku.edu.cn (J.L.); wangjie2015@hsc.pku.edu.cn (J.W.)

[†]These authors contributed equally to this work.

Table S1. Sequences of primers used for HBV DNA and HBV RNA quantification

Quantitative markers	Primers sequences (5'-3')
HBV DNA	F: CCGTCTGTGCCTTCTCATCTG
	R: AGTCCAAGAGTCCTCTTATGTAAGACCTT
HBV 3.5kb RNA	F: GAGTGTGGATTCGCACTCC
	R: GAGGCGAGGGAGTTCTTCT
ACTB	F: ACTGTGCCCATCTACGAGG
	R: CAGGCAGCTCGTAGCTCTT

F, forward; R, reverse.

Table S2. The positions of different HBV regions

Regions	Start position (nt)	End position (nt)
PreS1	2848	3204
NTCPbd	2884	3021
PreS2	3205	154
HBsAg	155	835
MHR	449	661
"a" determinant region	524	595
PreC	1814	1900
HBc	1901	2452
Polymerase	2307	1623
RT	130	1161
HBx	1374	1838
SPI	2718	2808
SPII	2983	3210
ENI	957	1361
XP	1171	1361
ENII	1685	1773
CP	1613	1849
BCP	1742	1849

Table S3. Sequences of primers used for plasmid construction

Primer names	Primers sequences (5’-3’)
sK47E-F	TCTAGGGGGAGCACCCGCGTGTCCTGGCCAAAA
sK47E-R	TTTTGGCCAGGACACGCGGGTGCTCCCCCTAGA
sP49L-F	GGAGCACCCACGTGTCTTGGCCAAAATTCGCAG
sP49L-R	CTGCGAATTTTGGCCAAGACACGTGGGTGCTCC

F, forward; R, reverse.

Table S4. HBV serologic results of each sample

ID	Group	Mothers				Infants at 7 months of age			Infants at 12 months of age		
		HBsAg ($\log_{10}$ IU/mL)	HBeAg ($\log_{10}$ S/CO)	HBV DNA ($\log_{10}$ IU/mL)	ALT (U/L)	Anti-HBs (mIU/mL)	Anti-HBc (S/CO)	HBV DNA ($\log_{10}$ IU/mL)*	Anti-HBs (mIU/mL)	Anti-HBc (S/CO)	HBV DNA ($\log_{10}$ IU/mL)*
M1	case	4.81	3.22	8.26	28.00	609.2	6.85	1.54	211.51	0.66	ND
M2	case	4.82	3.04	8.75	4.00	891.09	0.66	ND	957.43	0.09	1.25
M3	case	4.52	3.17	9.11	13.30	3697.53	8.24	ND	1769.15	1.07	3.2
M4	case	4.73	3.13	8.38	2.20	936.51	0.87	2.3	172.97	0.34	4.42
M5	case	4.69	3.15	8.55	13.8	609.89	5.45	ND	403.67	0.2	3.36
M6	case	4.37	3.15	8.79	37.8	12.72	0.1	1.24	26.56	3.4	ND
M7	case	4.59	3.19	8.15	15.00	821.92	6.53	ND	100.14	0.63	2.39
M8	case	4.42	3.23	8.62	16.30	1644.29	4.91	1.71	4794.68	1.06	ND
M9	case	4.56	3.08	8.64	19.00	957.6	NA	1.37	264.72	0.04	ND
M10	case	3.51	2.87	7.63	26.00	830.81	1.06	1.32	147.28	0.11	ND
M11	case	4.74	3.17	8.81	20.00	138.63	0.11	2.53	288.91	0.1	1.86
M12	case	4.43	1.34	8.13	8.00	2564.78	0.24	2.29	185.15	0.2	ND
M13	case	4.56	3.01	8.68	16.00	1609.38	1.41	ND	365.99	0.16	2.1
M14	case	3.21	3.12	6.57	23.00	1060.58	7.75	2.35	1139.38	1.42	ND
M15	case	4.16	3.23	8.31	17.00	2634.48	10.01	1.72	1596.1	2.85	2.91
M16	control	3.85	1.85	6.73	18.00	1699.53	6.94	ND	660.08	2.51	ND
M17	control	4.61	3.11	7.80	22.00	7754.51	NA	ND	2031.66	0.2	ND

M18	control	4.63	3.25	8.38	20.00	204.11	NA	ND	63.34	0.06	ND
M19	control	4.76	3.23	8.72	15.00	177.37	NA	ND	29.66	3.51	ND
M20	control	4.25	3.15	8.75	36.00	339.42	NA	ND	41.27	0.12	ND
M21	control	3.97	3.10	7.23	25.00	652.65	NA	ND	121.43	6.54	ND
M22	control	4.67	2.90	8.66	20.00	54.77	10.78	ND	24.34	4.25	ND
M23	control	4.45	3.24	8.09	26.00	1457.32	NA	ND	993.75	0.14	ND
M24	control	4.87	3.26	9.00	18.00	1854.97	NA	ND	335.31	0.14	ND
M25	control	4.28	3.05	8.41	23.00	881.01	1.59	ND	118.33	0.17	ND
M26	control	4.33	3.12	8.13	11.00	291.6	6.64	ND	51.21	0.5	ND
M27	control	4.66	3.18	8.66	14.00	800.85	0.07	ND	721.75	0	ND
M28	control	4.71	3.24	8.87	15.60	1809.06	0.98	ND	241.63	0.12	ND
M29	control	3.93	3.02	7.91	17.30	199.3	9.06	ND	197.3	0.25	ND
M30	control	3.36	2.45	6.61	15.10	359.23	8.12	ND	33.57	0.07	ND
M31	control	4.33	3.11	8.42	18.00	290.04	1.99	ND	220.13	0.17	ND
M32	control	3.72	1.42	6.17	37.00	1985.69	9.47	ND	564.1	0.91	ND
M33	control	4.25	3.08	7.79	17.20	285.04	6.95	ND	63.23	0.37	ND
M34	control	4.22	3.15	7.88	12.00	2843.7	0.11	ND	1172.98	0.11	ND
M35	control	3.20	3.15	7.55	17.00	2152.19	8.2	ND	179.49	0.29	ND
M36	control	4.86	3.03	8.92	17.30	1144.17	4.8	ND	266.31	0.38	ND
M37	control	4.35	3.03	8.12	13.00	544.04	1.5	ND	364.83	0.12	ND
M38	control	3.57	2.31	8.45	25.00	2902.58	4.11	ND	6268.01	0.75	ND

*HBV DNA titers marked in red represent corresponding serum samples were successfully sequenced by NGS.

NA, not available; ND, not detected.

Table S5. The characteristics of HBV quasispecies between the case and control groups of mothers

Characteristic	Case group	Control group	*P*
Full-length HBV genome_ntd	0.0011 (9e-04–0.0017)	0.0021 (0.0013–0.0026)	0.018
"a" determinant region_ntd	0.0011 (7e-04–0.0015)	0.0014 (8e-04–0.0073)	0.156
HBc_ntd	9e-04 (7e-04–0.0014)	0.0015 (0.001–0.0039)	0.021
MHR_ntd	9e-04 (8e-04–0.0015)	0.0019 (0.0011–0.0039)	0.013
NTCPbd_ntd	0.001 (7e-04–0.0018)	0.0019 (0.0013–0.0036)	0.0156
PreC_ntd	6e-04 (5e-04–9e-04)	7e-04 (5e-04–0.0011)	0.362
PreS1_ntd	0.0011 (7e-04–0.0014)	0.0018 (0.0013–0.0036)	0.013
PreS2_ntd	0.0011 (9e-04–0.0028)	0.0013 (0.0011–0.003)	0.238
RT_ntd	0.0011 (8e-04–0.0013)	0.0019(0.0011–0.0027)	0.013
HBsAg_ntd	0.0011 (7e-04–0.0012)	0.0016 (0.0011–0.0029)	0.019
XP_ntd	0.0014 (9e-04–0.0022)	0.0018 (0.0014–0.0037)	0.148
HBx_ntd	0.0013 (0.0011–0.0022)	0.0019 (0.0015–0.0038)	0.081
BCP_ntd	6e-04 (5e-04–0.001)	0.001 (7e-04–0.0015)	0.066
CP_ntd	0.0012 (8e-04–0.0022)	0.0019 (0.0013–0.0046)	0.124
EnI_ntd	0.0011 (9e-04–0.0017)	0.0018 (0.0013–0.0033)	0.054
EnII_ntd	0.0011 (0.001–0.0025)	0.0017 (0.0013–0.0033)	0.117
SPI_ntd	9e-04 (7e-04–0.0012)	0.0013 (9e-04–0.0037)	0.104
SPII_ntd	0.0012 (9e-04–0.0015)	0.002 (0.0013–0.0035)	0.026
"a" determinant_aad	0.0595 (0.0543–0.0972)	0.1002 (0.0606–0.1603)	0.0833
HBc_aad	0.1056 (0.1018–0.1274)	0.1539 (0.1212–0.1744)	0.0041
MHR_aad	0.1708 (0.1425–0.244)	0.2345 (0.1693–0.3026)	0.0423
NTCPbd_aad	0.1307 (0.1226–0.1679)	0.1889 (0.1492–0.2844)	0.006
PreC_aad	0.0764 (0.0595–0.0994)	0.1435 (0.1103–0.1798)	0.0001
PreS1_aad	0.2097 (0.1816–0.2366)	0.2827 (0.2025–0.3592)	0.1135
PreS2_aad	0.0765 (0.0574–0.1221)	0.115 (0.0907–0.1936)	0.0393
RT_aad	0.2855 (0.2596–0.3231)	0.3107 (0.2896–0.366)	0.0599

HBsAg_aad	0.2672 (0.2406–0.3405)	0.3482 (0.2518–0.4607)	0.2097
HBx_aad	0.1323 (0.116–0.1394)	0.1666 (0.1387–0.2463)	0.0055
"a" determinant_*dN*	8e-04 (5e-04–0.0011)	0.0011 (4e-04–0.003)	0.362
HBc_*dN*	7e-04 (6e-04–0.0014)	0.0011 (9e-04–0.0018)	0.041
MHR_*dN*	7e-04 (6e-04–0.001)	0.0014 (0.001–0.0037)	0.015
NTCPbd_*dN*	9e-04 (6e-04–0.001)	0.0013 (0.001–0.0022)	0.005
PreC_*dN*	6e-04 (3e-04–9e-04)	6e-04 (4e-04–0.0013)	0.446
PreS1_*dN*	0.0012 (8e-04–0.0013)	0.0017 (0.0012–0.0029)	0.05
PreS2_*dN*	0.001 (7e-04–0.0021)	0.0011 (8e-04–0.0024)	0.483
RT_*dN*	7e-04 (7e-04–0.0011)	0.0016 (9e-04–0.0022)	0.016
HBsAg_*dN*	8e-04 (6e-04–0.0011)	0.0012 (8e-04–0.002)	0.044
HBx_*dN*	0.001 (9e-04–0.0015)	0.0012 (9e-04–0.0024)	0.8
"a" determinant_*dS*	0.0011 (9e-04–0.0014)	0.0016 (6e-04–0.0024)	0.731
HBc_*dS*	0.0011 (8e-04–0.0014)	0.0011 (7e-04–0.0018)	0.777
MHR_*dS*	8e-04 (7e-04–0.0012)	0.0013 (8e-04–0.0024)	0.204
NTCPbd_*dS*	0.0014 (0.001–0.0016)	0.0025 (0.0017–0.005)	0.0132
PreC_*dS*	6e-04 (4e-04–0.0013)	8e-04 (0–0.0012)	0.868
PreS1_*dS*	0.0013 (8e-04–0.0015)	0.0014 (6e-04–0.0026)	0.446
PreS2_*dS*	0.0018 (0.001–0.002)	0.0012 (8e-04–0.0032)	0.687
RT_*dS*	8e-04 (7e-04–0.001)	0.0013 (0.001–0.0043)	0.008
HBsAg_*dS*	0.001 (8e-04–0.0015)	0.0013 (0.001–0.0021)	0.139
HBx_*dS*	0.0014 (9e-04–0.0019)	0.0016 (0.0012–0.0036)	0.25
Full-length HBV genome_nts	0.8945 (0.843–0.953)	0.9729 (0.8941–0.9931)	0.086
"a" determinant_nts	0.0859 (0.0559–0.1202)	0.1194 (0.0775–0.1857)	0.148
HBc_nts	0.3966 (0.3401–0.461)	0.4802 (0.3911–0.5846)	0.086
MHR_nts	0.2283 (0.1459–0.2753)	0.2637 (0.1891–0.3896)	0.289
NTCPbd_nts	0.1273 (0.116–0.1516)	0.1855 (0.1338–0.2728)	0.0315
PreC_nts	0.104 (0.0932–0.1219)	0.1116 (0.1007–0.1401)	0.332
PreS1_nts	0.2931 (0.2815–0.3459)	0.4197 (0.3213–0.5175)	0.058

PreS2_nts	0.1481 (0.1292–0.2306)	0.2021 (0.1518–0.2394)	0.124
RT_nts	0.5583 (0.5192–0.7171)	0.7113 (0.574–0.8062)	0.124
HBsAg_nts	0.4511 (0.3952–0.5833)	0.5732 (0.4397–0.6619)	0.132
XP_nts	0.2105 (0.1584–0.2669)	0.2488 (0.1887–0.3212)	0.184
HBx_nts	0.4696 (0.3666–0.538)	0.5207 (0.425–0.6899)	0.132
BCP_nts	0.2108 (0.1434–0.2713)	0.242 (0.1815–0.3703)	0.276
CP_nts	0.3309 (0.2311–0.3828)	0.3736 (0.2768–0.5287)	0.204
EnI_nts	0.3333 (0.2894–0.4304)	0.3903 (0.3035–0.5122)	0.184
EnII_nts	0.1857 (0.1037–0.2361)	0.2288 (0.1417–0.3517)	0.165
SPI_nts	0.0729 (0.0628–0.0967)	0.1 (0.0758–0.1448)	0.058
SPII_nts	0.2049 (0.1913–0.2484)	0.2682 (0.2333–0.3618)	0.03
"a" determinant_aas	0.0694 (0.0508–0.1032)	0.1025 (0.0678–0.1759)	0.165
HBc_aas	0.3189 (0.2685–0.3904)	0.3798 (0.3111–0.5035)	0.132
MHR_aas	0.1817 (0.1243–0.248)	0.2227 (0.1527–0.3576)	0.226
NTCPbd_aas	0.1058 (0.0925–0.1189)	0.1473 (0.1013–0.2061)	0.0887
PreC_aas	0.0949 (0.0833–0.1121)	0.0969 (0.0905–0.1181)	0.56
PreS1_aas	0.2482 (0.2326–0.2973)	0.3471 (0.2445–0.4596)	0.066
PreS2_aas	0.1249 (0.1039–0.1912)	0.1654 (0.1238–0.1892)	0.165
RT_aas	0.4198 (0.3843–0.5895)	0.5389 (0.4516–0.6292)	0.092
HBsAg_aas	0.3913 (0.3526–0.5431)	0.5311 (0.3802–0.5998)	0.139
HBx_aas	0.4067 (0.3093–0.4582)	0.4358 (0.3581–0.6206)	0.11

aad, genetic distances at the amino acid level; aas, Shannon Entropy at aminoacid level; *dN*, genetic distance of nonsynomynous substitution; *dS*, genetic distance of synomynous substitution; ntd, genetic distances at the nucleotide level; nts, Shannon entropy at the nucleotide level.

Table S6. The AUC and cut-off value of quasispecies characteristics in predicitng the risk of OBI in infants born to mothers with chronic HBV infection

Quasispecies characteristics	AUC (95%CI)	*p* value	Threshold	Specificity	Sensitivity
Full-length HBV genome_ntd	0.73 (0.558–0.903)	0.018	0.0012713	0.667	0.783
PreS1_ntd	0.742 (0.573–0.911)	0.013	0.0012389	0.667	0.783
NTCPbd_ntd	0.736(0.567–0.906)	0.016	0.0012603	0.667	0.739
HBsAg_ntd	0.728 (0.554–0.901)	0.02	0.0012953	0.8	0.652
MHR_ntd	0.742 (0.575–0.909)	0.013	0.0008909	0.533	0.913
HBc_ntd	0.725 (0.549–0.901)	0.021	0.000843	0.467	0.913
RT_ntd	0.742 (0.572–0.912)	0.013	0.0015341	0.867	0.609
SPII_ntd	0.716 (0.542–0.89)	0.027	0.001295	0.667	0.739
Pres2_aad	0.701 (0.51–0.893)	0.039	0.0808075	0.6	0.87
NTCPbd_aad	0.768 (0.611–0.926)	0.006	0.1311756	0.533	0.913
MHR_aad	0.699 (0.522–0.875)	0.042	0.1543295	0.4	0.957
HBc_aad	0.78 (0.623–0.936)	0.004	0.1345506	0.867	0.696
PreC_aad	0.881 (0.769–0.993)	<0.001	0.1055846	0.867	0.783
HBx_aad	0.771 (0.614–0.928)	0.005	0.1418379	0.8	0.739
NTCPbd_*dN*	0.774 (0.611–0.936)	0.005	0.0010485	0.8	0.739
HBsAg_*dN*	0.696 (0.52–0.872)	0.045	0.0011994	0.867	0.478
MHR_*dN*	0.736 (0.568–0.904)	0.016	0.00113	0.8	0.696
HBc_*dN*	0.699 (0.508–0.889)	0.042	0.0007319	0.6	0.913
RT_*dN*	0.733 (0.565–0.901)	0.017	0.0010938	0.8	0.652
NTCPbd_*dS*	0.742(0.568–0.916)	0.013	0.0015233	0.733	0.826
RT_*dS*	0.757 (0.594–0.919)	0.009	0.0010616	0.8	0.739
NTCPbd_nts	0.71(0.531–0.889)	0.032	0.134862	0.667	0.739
SPII_nts	0.71 (0.528–0.892)	0.032	0.2313339	0.733	0.783
Proportion of Seq_type7 (PreC)	0.98 (0.946–1)	<0.001	0.87%	1	0.913
Proportion of Seq_type8 (PreC)	0.817 (0.675–0.96)	0.001	0.19%	0.867	0.783
Proportion of Seq_type9 (PreC)	0.98 (0.942–1)	<0.001	1.04%	0.933	0.957

Proportion of Seq_type7to9 (PreC)	1 (1–1)	<0.001	2.05%	1	1
HBV DNA level	0.622 (0.437–0.807)	0.21	8.125	0.867	0.478

aad, genetic distances at the amino acid level; *dN*, genetic distance of nonsynomynous substitution; *dS*, genetic distance of synomynous substitution; ntd, genetic distances at the nucleotide level.

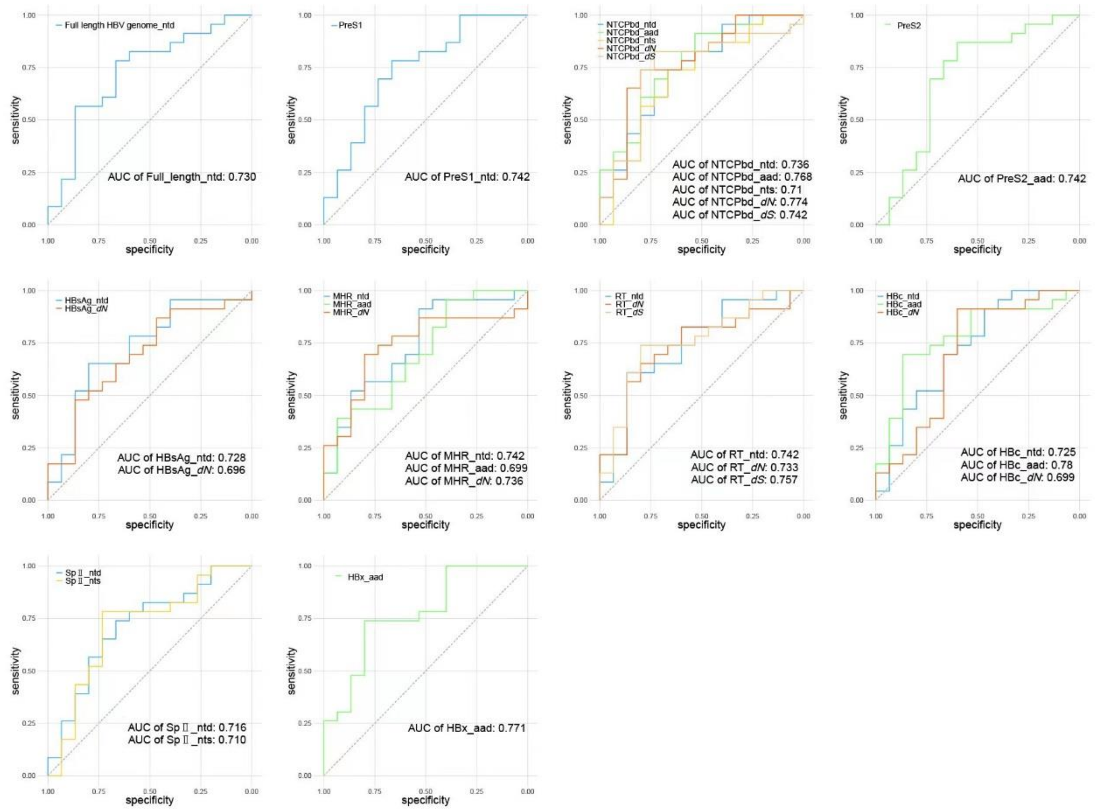

Figure S1. The values of quasispecies characteristics and the proportion of PreC seq_type 1–3 that were significantly different between the two groups in predicting the risk of infant OBI were explored by receiver operating characteristic (ROC) curve. aad, genetic distances at the amino acid level; AUC, area under the ROC curve; *dN*, genetic distance of non-synonymous substitution; *dS*, genetic distance of synonymous substitution; ntd, genetic distances at the nucleotide level; nts, Shannon entropy at the nucleotide level.

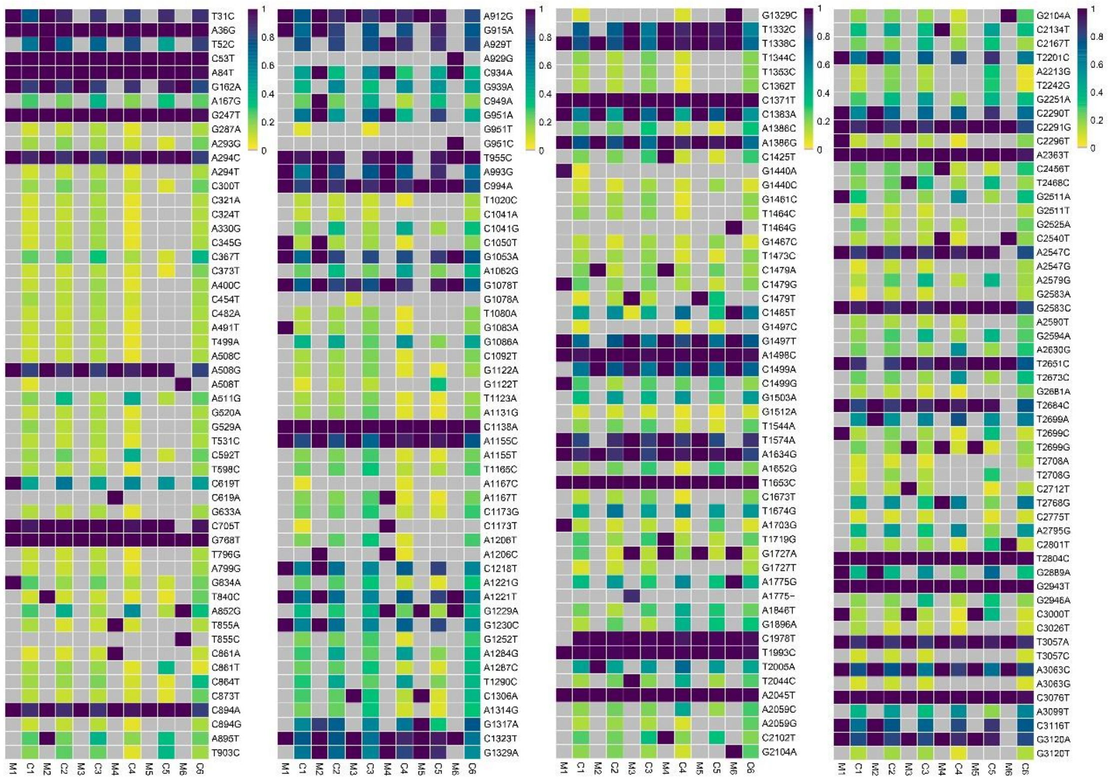

Figure S2. 119 amino acid substitutions among 473 mutations detected at 369 positions with increased complexity in OBI infants.
